# Constitutively activated PI3K accelerates tumor initiation and modifies histopathology of breast cancer

**DOI:** 10.1038/oncsis.2016.65

**Published:** 2016-10-31

**Authors:** M R Sheen, J D Marotti, M J Allegrezza, M Rutkowski, J R Conejo-Garcia, S Fiering

**Affiliations:** 1 Department of Microbiology and Immunology, Geisel School of Medicine at Dartmouth, Lebanon, NH, USA; 2 Department of Pathology and Laboratory Medicine, Dartmouth-Hitchcock Medical Center, Lebanon, NH, USA; 3 Geisel School of Medicine at Dartmouth, Hanover, NH, USA; 4 Norris Cotton Cancer Center, Lebanon, NH, USA; 5 Tumor Microenvironment and Metastasis Program, The Wistar Institute, Philadelphia, PA, USA; 6 Department of Genetics, Geisel School of Medicine at Dartmouth, Hanover, NH, USA

## Abstract

The gene encoding phosphatidylinositol 3-kinase catalytic subunit α-isoform (*PIK3CA*, *p110α*) is frequently activated by mutation in human cancers. Based on detection in some breast cancer precursors, *PIK3CA* mutations have been proposed to have a role in tumor initiation. To investigate this hypothesis, we generated a novel mouse model with a Cre-recombinase regulated allele of *p110α* (myristoylated-*p110α,* myr-*p110α*) along with *p53*^*fl/fl*^ deletion and *Kras*^*G12D*^ also regulated by Cre-recombinase. After instillation of adenovirus-expressing Cre-recombinase into mammary ducts, we found that myr-*p110α* accelerated breast tumor initiation in a copy number-dependent manner. Breast tumors induced by *p53*^*fl/fl*^;*Kras*^*G12D*^ with no or one copy of myr-*p110α* had predominantly sarcomatoid features, whereas two copies of myr-*p110α* resulted in tumors with a carcinoma phenotype. This novel model provides experimental support for importance of active p110α in breast tumor initiation, and shows that the amount of PI3K activity can affect the rate of tumor initiation and modify the histological phenotype of breast cancer.

## Introduction

Receptor tyrosine kinase-phosphatidylinositol 3-kinase (PI3K) signaling is a central integrator of metabolism, cell growth and cell survival, and deregulated PI3K signaling increases tumorigenicity.^1, 2^ Cancer-associated mutations occur in several components of the pathway including activating mutations of both the PI3K catalytic subunit α-isoform gene (*PIK3CA*, *p110α*)^3^ and the downstream signaling molecule *AKT1*,^4^ as well as deletion of the negative pathway regulator, *PTEN*.^5^ These alterations all result in increased activity of the PI3K signaling pathway. The significance of *p110α* in cancer is demonstrated by the high frequency of activating mutations in many common human cancers that increase the catalytic activity of PI3K.^3, 6^

High-throughput RNA sequencing and tumor resequencing have revealed that hyperactivating mutations in the PI3K signaling pathway occur in a substantial percentage of breast cancers.^7^ The *PIK3CA* gene itself, encoding the p110α catalytic subunit, is the most frequently mutated gene in breast cancer,^8, 9^ with mutations in 25–40% of all breast cancers.^10, 11^ However, the prognostic implications of *p110α* mutations remain unclear. Although the association of PI3K mutations with poor prognosis has been reported,^12, 13^ others have described a correlation with improved outcome.^14, 15^

Whole-exome analysis of finely dissected and matched mammary tumors has shown that alterations in the *p110α* gene are detected at the same frequencies in ductal carcinoma *in situ* (DCIS), DCIS adjacent to invasive carcinoma and invasive carcinoma.^16^ In addition, the *p110α* sequences are often found to be identical in invasive and *in situ* areas of the same breast tumor.^16^ This pattern of mutation suggests that *p110α* mutations are a relatively early event in breast cancer development. In light of these findings, mutational activation of the PI3K signaling pathway has been proposed to have a role in breast tumor initiation.

Multiple mouse models of the PI3K pathway-driven cancer have recently been developed to investigate the impact of PI3K mutation or combination of other related mutations on breast cancer development under the control of MMTV LTR or WAP promoter.^17, 18, 19, 20, 21^ However, the role of PI3K activation in tumor initiation, progression and phenotype is not well understood. In the present study, the PI3K catalytic subunit p110α was myristoylated to force localization to the cell membrane, which is sufficient to release its kinase activity from normal regulation, and to thus activate the PI3K signaling pathway. The expression of myristoylated-*p110α* was regulated by Cre-recombinase expressed from Ad-Cre virus, which allowed for spatial and temporal experimental control. To ascertain the impact of *p110α* activating mutations in tumor initiation and progression, we combined myr-*p110α* with frequently co-occurring mutations of two genes, *p53* and *Kras*, to generate novel models of breast, lung and potentially other cancers whose induction is controlled by the researcher.

## Results

### *In vivo* expression of myr-*p110α* and homozygous *p53* deletion in milk duct causes slowly developing mammary tumors

To elucidate the role of activating mutations of *p110α* in tumorigenesis, we engineered a mouse with conditional myristoylated*-p110α* (myr-*p110α*) ubiquitously expressed from the *Rosa26* locus (Supplementary Figure 1).^22^ Constitutive expression of myr-*p110α* during development was embryonic lethal.^22^ To have temporal and spatial control over tumorigenic genetic changes in breast cancer, we utilized *in vivo* administration of adenovirus-expressing Cre-recombinase (Ad-Cre) into the mammary duct of virgin females.^23^

**Figure 1:**
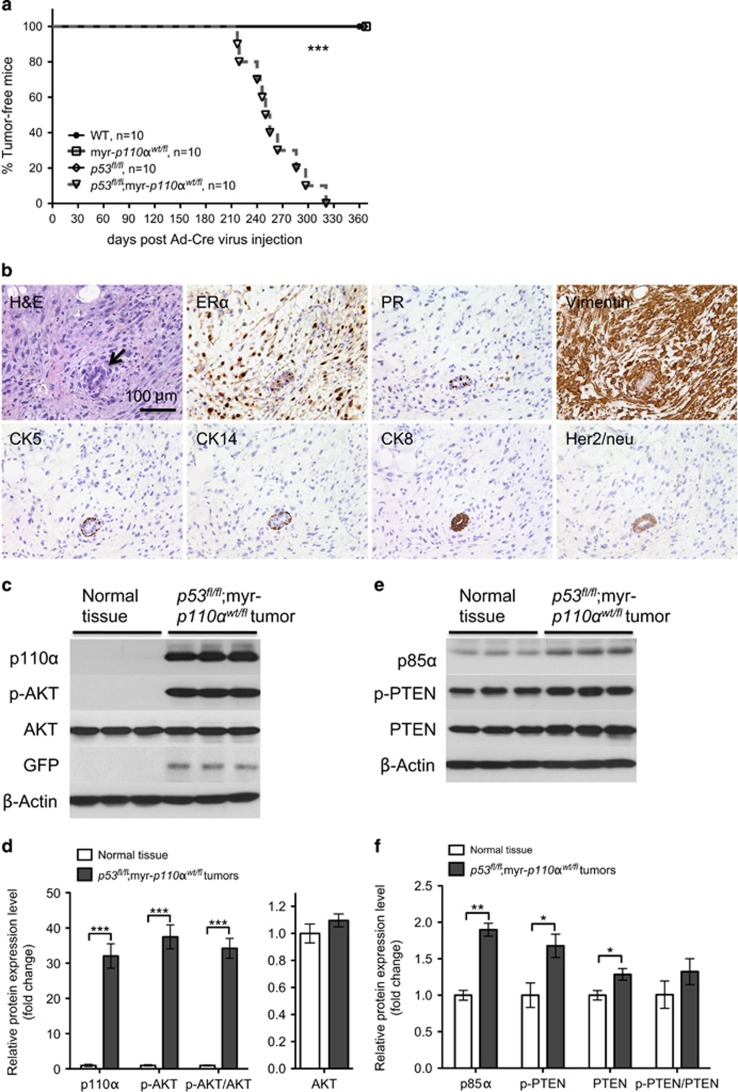
*In vivo* expression of myr-*p110α* and homozygous *p53* deletion in milk duct activates PI3K signaling and develops mammary tumors. (**a**) Kaplan–Meier survival curve demonstrating breast tumor onset defined as the first palpation-mediated recognition of tumors (*n*=10 per group). Log-rank (Mantel–Cox) test was used for statistical analysis. ***P<0.001. (**b**) Hematoxylin and eosin (H&E) staining and immunohistochemistry of mammary tumors from *p53*^*fl/fl*^;myr-*p110α*^*wt/fl*^ females stained with antibodies against following markers: ERα (estrogen receptor α), PR (progesterone receptor), vimentin (mesenchymal marker), CK5 (basal marker), CK14 (myoepithelial marker), CK8 (luminal marker) and Her2/neu. Representative pictures of H&E staining and immunostaining were taken with × 200 magnification. Scale bar 100 μm indicates scale for all images. The poorly differentiated neoplasm is immunoreactive with ERα and vimentin, and negative for the remaining antigens. A normal duct serves as an internal control (arrow in H&E). (**c**) Western blot analysis of p110α, p-AKT, AKT and GFP with normal mammary tissue and mammary tumors from *p53*^*fl/fl*^;myr-*p110α*^*wt/fl*^ mice. (**d**) The graphs of western blot signals of p110α, p-AKT and p-AKT/AKT ratio quantified and normalized with respect to β-actin. (**e**) Western blot analysis of p85α, p-PTEN and PTEN with wild-type mammary tissue and *p53*^*fl/fl*^;myr-*p110α*^*wt/fl*^ mammary tumors. (**f**) The graphs of western blot signals of p85α, p-PTEN, PTEN and p-PTEN/PTEN ratio quantified and normalized with respect to β-actin. For **d** and **f**, mean fold increase compared with mammary tissue lysates from a non-Cre harboring age-matched female was calculated after normalization. Error bars are the mean±s.e.m. Two-tailed unpaired Student's *t-*test was used for statistical analysis. *P<0.05, **P<0.01 and ***P<0.001. Data in **c**–**f** are representative of four independent experiments with biological and technical replicates.

As activated myr-*p110α* by itself had minimal transforming activity (Supplementary Figure 2) and the *p53* gene is the second most frequently mutated gene in breast cancer,^8, 24^ we assessed the effect of myr-*p110α* and homozygous *p53* deletion on breast tumor development. In line with its tumorigenic potential in mouse embryonic fibroblasts (MEFs; Supplementary Figure 2) and previous studies showing the cooperation between *Pik3ca*^*H1047R*^ activating mutation and *p53* knockout *in vivo*,^17, 20^ mice expressing one copy of myr-*p110α* and homozygous for *p53* deletion (*p53*^*fl/fl*^;myr-*p110α*^*wt/fl*^) developed mammary tumors with 100% penetrance but with a long latency (mean tumor onset 259.6±10.6 days; Figure 1a). Histological analysis of the tumors revealed predominantly poorly differentiated or dedifferentiated malignant neoplasms with sarcomatoid features as well as small areas of high-grade carcinoma. The sarcomatoid neoplasms were positive for estrogen receptor α (ERα), focal progesterone receptor (PR) and vimentin (mesenchymal marker); they were negative for cytokeratin 5 (CK5, basal epithelial marker), cytokeratin 14 (CK14, myoepithelial marker), cytokeratin 8 (CK8, luminal epithelial marker) and human epidermal growth factor receptor 2 (Her2/neu; Figure 1b). In contrast, mice harboring either myr-*p110α*^*wt/fl*^ or homozygous *p53* deletion only, failed to form mammary tumors during 12 months of observation.

### Mammary tumors expressing *p53*^*fl/fl*^;myr-*p110α*^*wt/fl*^ activate PI3K signaling

Increased PI3K signaling activates AKT, a key downstream effector of PI3K signaling pathway.^25^ To test for PI3K signaling activation, mammary tumors were analyzed for levels of total p110α, total AKT, the active phosphorylated form of AKT (p-AKT), PI3K regulatory subunit p85α, PI3K negative regulator PTEN and the less active phosphorylated form of PTEN with reduced lipid phosphatase activity (p-PTEN).^26, 27^ As compared with normal mammary tissue, mammary tumors from *p53*^*fl/fl*^;myr-*p110α*^*wt/fl*^ females had elevated levels of total p110α (32-fold), p-AKT (37-fold) and p-AKT/AKT ratio (34-fold; Figures 1c and d). Expression of myr-*p110α* was confirmed by the detection of GFP in mammary tumors (Figure 1c). The *p53*^*fl/fl*^;myr-*p110α*^*wt/fl*^ mammary tumors also displayed significantly increased total protein levels of p85α (1.9-fold), PTEN (1.3-fold) and p-PTEN (1.7-fold; Figures 1e and f), suggesting naturally occurring negative feedback mechanisms to inhibit abnormal PI3K activity driven by myr-*p110α*. Elevated levels of p-AKT were associated with the increased PI3K activity despite increased levels of PI3K regulators, p85α and PTEN, demonstrating ineffective regulation by increased p85α and PTEN.

### Addition of myr-*p110α* into *p53*^*fl/fl*^;*Kras^G12D^* double mutants accelerates mammary tumor initiation but not tumor growth rate

**Figure 2:**
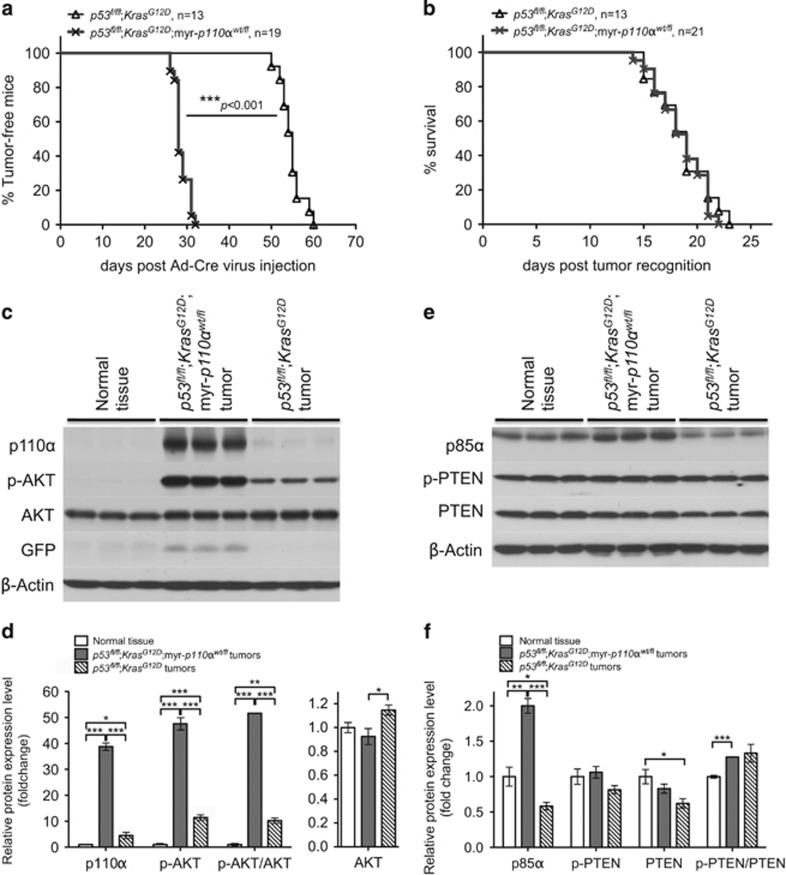
Addition of myr-*p110α* into *p53*^*fl/fl*^;*Kras*^*G12D*^ double mutants increases PI3K signaling and accelerates mammary tumor initiation with no impact on tumor growth rate. (**a**) Kaplan–Meier survival curve demonstrating breast tumor onset in the two strains. Log-rank (Mantel–Cox) test was used for statistical analysis. ***P<0.001. (**b**) Kaplan–Meier survival curve demonstrating mammary tumor progression in each strain. The period of tumor growth was defined as the number of days between when a mammary tumor was first observed in the size range of 12–15 mm^2^ and when tumor surface area reached the end point of 100 mm^2^. Log-rank (Mantel–Cox) test was used for statistical analysis. *P*>0.05, non-significant. (**c**) Western blot analysis of p110α, p-AKT, AKT and GFP with wild-type (WT) mammary tissue and mammary tumors from *p53*^*fl/fl*^;*Kras*^*G12D*^ and *p53*^*fl/fl*^;*Kras*^*G12D*^;myr-*p110α*^*wt/fl*^ mice. (**d**) The graphs of western blot signals of p110α, p-AKT, p-AKT/AKT ratio and AKT quantified and normalized with respect to β-actin. (**e**) Western blot analysis of p85α, p-PTEN and PTEN with WT mammary tissue and mammary tumors from *p53*^*fl/fl*^;*Kras*^*G12D*^ and *p53*^*fl/fl*^;*Kras*^*G12D*^;myr-*p110α*^*wt/fl*^ mice. (**f**) The graphs of western blot signals of p85α, p-PTEN, PTEN and p-PTEN/PTEN ratio quantified and normalized with respect to β-actin. Mean fold increase compared with mammary tissue lysates from a non-Cre harboring age-matched female was calculated after normalization. Error bars are the mean±s.e.m. Two-tailed unpaired Student's *t-*test was used for statistical analysis. *P<0.05, **P<0.01 and ***P<0.001. Data in **c**–**f** are representative of four independent experiments with biological and technical replicates.

It has been proposed that *PIK3CA* has a role in tumor initiation, as its mutations are detected in precursors of breast cancer.^28^ To investigate the role of myr-*p110α* in tumor initiation and progression, we exploited the Cre-inducible oncogenic mouse model *p53*^*fl/fl*^;*Kras*^*G12D*^, which harbors a homozygous *p53* deletion and a *Kras*^*G12D*^ activating missense allele that rapidly grows tumors.^29^ Based on the published breast cancer genomics data, *Kras*^*G12D*^ mutation tends to co-occur with *p53* mutations (Supplementary Figures 3A and 3B). To assess tumor initiation and development in *p53*^*fl/fl*^;*Kras*^*G12D*^ mice in the absence or presence of myr-*p110α*, mice harboring *p53*^*fl/fl*^;*Kras*^*G12D*^ or *p53*^*fl/fl*^;*Kras*^*G12D*^;myr-*p110α*^*wt/fl*^ were generated. Ad-Cre virus was administered into the milk duct of virgin females and mice were monitored for initial tumor formation and growth rate. Both *p53*^*fl/fl*^;*Kras*^*G12D*^ and *p53*^*fl/fl*^;*Kras*^*G12D*^;myr-*p110α*^*wt/fl*^ mice developed mammary tumors with 100% penetrance. Notably, although *p53*^*fl/fl*^;*Kras*^*G12D*^ mice began to form palpable mammary tumors (tumor size ranges 12–15 mm^2^) with a mean tumor onset of 54.8±0.7 days, *p53*^*fl/fl*^;*Kras*^*G12D*^;myr-*p110α*^*wt/fl*^ mice started to form palpable mammary tumors with a mean tumor onset of 28.7±0.4 days (Figure 2a), indicating roughly two-fold acceleration of tumor initiation due to expression of myr-*p110α*.

To examine the difference in tumor progression, the period of tumor growth was defined as the number of days between when a mammary tumor was first observed and when the animal reached end point, and depicted in the Kaplan–Meier survival curve. Although initiation was markedly accelerated, once the tumor was established, there was no difference in tumor growth rate between *p53*^*fl/fl*^;*Kras*^*G12D*^ and *p53*^*fl/fl*^;*Kras*^*G12D*^;myr-*p110α*^*wt/fl*^ mice. Mammary tumors from *p53*^*fl/fl*^;*Kras*^*G12D*^ mice reached end point with a mean of 18.7±0.7 days, and *p53*^*fl/fl*^;*Kras*^*G12D*^;myr-*p110α*^*wt/fl*^ mammary tumors reached end point with a mean of 18.4±0.5 days (Figure 2b).

### Mammary tumors from *p53^fl/fl^;Kras^G12D^*;myr-*p110α^wt/fl^* have higher p-AKT levels than those from *p53^fl/fl^*;*Kras^G12D^*

To biochemically characterize mammary tumors from *p53*^*fl/fl*^;*Kras*^*G12D*^ and *p53*^*fl/fl*^;*Kras*^*G12D*^;myr-*p110α*^*wt/fl*^, we tested the PI3K pathway activation status of the mammary tumors by analyzing the levels of p110α, AKT, p-AKT, p85α, PTEN and p-PTEN.

Mammary tumors from *p53*^*fl/fl*^;*Kras*^*G12D*^ females had significantly elevated levels of total p110α (4.5-fold), p-AKT (11-fold) and p-AKT/AKT ratio (10-fold) as shown in Figures 2c and d, in comparison with normal mammary tissue. *p53*^*fl/fl*^;*Kras*^*G12D*^;myr-*p110α*^*wt/fl*^ mammary tumors with one copy of myr-*p110α* had further elevated expression levels of total p110α (39-fold), and this resulted in increased p-AKT levels by 48-fold and increased p-AKT/AKT ratio (52-fold; Figures 2c and d). Expression of myr-*p110α* via Ad-Cre virus injection was confirmed by GFP expression in *p53*^*fl/fl*^;*Kras*^*G12D*^;myr-*p110α*^*wt/fl*^ mammary tumors (Figure 2c).

**Figure 3:**
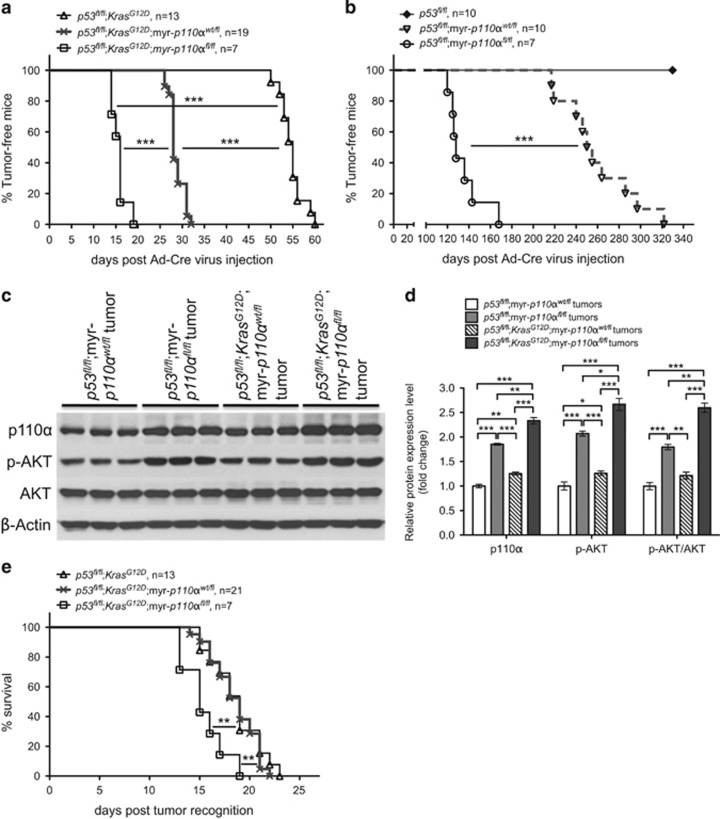
Two copies of myr-*p110α* further increase PI3K activity and accelerate mammary tumor initiation and growth rate. (**a**) Kaplan–Meier survival curve demonstrating the breast tumor onset in three strains: *p53*^*fl/fl*^;*Kras*^*G12D*^, *p53*^*fl/fl*^;*Kras*^*G12D*^;myr-*p110α*^*wt/fl*^ and *p53*^*fl/fl*^;*Kras*^*G12D*^;myr-*p110α*^*fl/fl*^. (**b**) Kaplan–Meier survival curve demonstrating the breast tumor onset in three strains: *p53*^*fl/fl*^, *p53*^*fl/fl*^;myr-*p110α*^*wt/fl*^ and *p53*^*fl/fl*^;myr-*p110α*^*fl/fl*^. (**c**) Western blot analysis of p110α, p-AKT and AKT with mammary tumors from *p53*^*fl/fl*^;myr-*p110α*^*wt/fl*^, *p53*^*fl/fl*^;myr-*p110α*^*fl/fl*^, *p53*^*fl/fl*^;*Kras*^*G12D*^;myr-*p110α*^*wt/fl*^ and *p53*^*fl/fl*^;*Kras*^*G12D*^;myr-*p110α*^*fl/fl*^ mice. (**d**) The graphs of western blot signals of p110α, p-AKT and p-AKT/AKT ratio quantified and normalized with respect to β-actin. Mean fold increase compared with *p53*^*fl/fl*^;myr-*p110α*^*wt/fl*^ mammary tumors was calculated after normalization. Error bars are the mean±s.e.m. Two-tailed unpaired Student's *t-*test was used for statistical analysis. *P<0.05, **P<0.01 and ***P<0.001. Data in **c** and **d** are representative of three independent experiments with biological and technical replicates. (**e**) Kaplan–Meier survival curve demonstrating mammary tumor progression in three strains: *p53*^*fl/fl*^;*Kras*^*G12D*^, *p53*^*fl/fl*^;*Kras*^*G12D*^;myr-*p110α*^*wt/fl*^ and *p53*^*fl/fl*^;*Kras*^*G12D*^;myr-*p110α*^*fl/fl*^. The period of tumor growth was defined as the number of days between when a mammary tumor was first observed in the size range of 12–15 mm^2^ and when tumor size reached 100 mm^2^. For **a**, **b** and **e**, Log-rank (Mantel–Cox) test was used for statistical analysis with **P<0.01 and ***P<0.001. To determine whether copy number increase in myr-*p110α* would have further impact on tumor initiation and progression in breast cancer models, the data generated from *p53*^*fl/fl*^;*Kras*^*G12D*^ and *p53*^*fl/fl*^;*Kras*^*G12D*^;myr-*p110α*^*wt/fl*^ females shown in the Figures 2a and b, and the data generated from *p53*^*fl/fl*^ and *p53^fl/fl^*;myr-*p110α^wt/fl^* females shown in the Figure 1a, were replicated for comparison.

Mammary tumors from *p53*^*fl/fl*^;*Kras*^*G12D*^;myr-*p110α*^*wt/fl*^ mice had elevated accumulation of regulatory subunit p85α by two-fold as compared with normal mammary tissue, whereas *p53*^*fl/fl*^;*Kras*^*G12D*^ mammary tumors displayed significantly decreased p85α level (0.6-fold) as shown in Figures 2e and f. The PTEN levels had a modest but statistically significant 0.6-fold decrease in *p53*^*fl/fl*^;*Kras*^*G12D*^ mammary tumors compared with normal mammary tissue (Figures 2e and f). The ratios of p-PTEN/PTEN were slightly but significantly increased in *p53*^*fl/fl*^;*Kras*^*G12D*^;myr-*p110α*^*wt/fl*^ (1.3-fold) mammary tumors (Figure 3f), indicating overall increase of less active p-PTEN.

### Two copies of myr-*p110α* further accelerate mammary tumor initiation and promote tumor progression

To determine how doubling the copy number of myr-*p110α* would impact tumorigenesis in this breast cancer model, we generated homozygous mice with two copies of myr-*p110α* transgene along with *p53*^*fl/fl*^;*Kras*^*G12D*^ mutants (*p53*^*fl/fl*^;*Kras*^*G12D*^;myr-*p110α*^*fl/fl*^). After Ad-Cre virus administration into the milk duct of virgin females, the time of tumor onset and tumor growth rate of *p53*^*fl/fl*^;*Kras*^*G12D*^;myr-*p110α*^*fl/fl*^ mammary tumors were compared with those of *p53*^*fl/fl*^;*Kras*^*G12D*^ and *p53*^*fl/fl*^;*Kras*^*G12D*^;myr-*p110α*^*wt/fl*^ mammary tumors to assess the effect of homozygous myr-*p110α* alleles.

Intraductal delivery of Ad-Cre virus into *p53*^*fl/fl*^;*Kras*^*G12D*^;myr-*p110α*^*fl/fl*^ mice induced the development of mammary tumors with 100% penetrance and a mean tumor onset of 15.7±0.6 days (Figure 3a). This tumor initiation was much faster than *p53*^*fl/fl*^;*Kras*^*G12D*^;myr-*p110α*^*wt/fl*^ mice (one copy of myr-*p110α*, 28.7±0.4 days) and *p53*^*fl/fl*^;*Kras*^*G12D*^ mice (zero copy of myr-*p110α*, 54.8±0.7 days). In accordance with this result, addition of two copies of myr-*p110α* into homozygous *p53* deletion (*p53*^*fl/fl*^;myr-*p110α*^*fl/fl*^) also facilitated mammary tumor initiation compared with *p53*^*fl/fl*^;myr-*p110α*^*wt/fl*^ (135.1±6.2 days vs 259.6±10.6 days; Figure 3b).

To check whether there would be a correlation between the extent of PI3K signaling activation and the rate of tumor initiation, we evaluated the PI3K signaling activation status by comparing the levels of p-AKT between *p53*^*fl/fl*^;myr-*p110α*^*wt/fl*^, *p53*^*fl/fl*^;myr-*p110α*^*fl/fl*^, *p53*^*fl/fl*^;*Kras*^*G12D*^;myr-*p110α*^*wt/fl*^ and *p53*^*fl/fl*^;*Kras*^*G12D*^;myr-*p110α*^*fl/fl*^ mammary tumors. Two copies of myr-*p110α* significantly elevated levels of total p110α (1.9-fold) and p-AKT (2.1-fold), as compared with one copy of myr-*p110α* (Figures 3c and d). We also tested the extent of PI3K signaling activation in mammary tumors harboring zero, one or two copies of myr-*p110α* transgene combined with either *p53*^*fl/fl*^ or *p53*^*fl/fl*^;*Kras*^*G12D*^ mutants. The levels of PI3K signaling activation were significantly elevated in a myr-*p110α* copy number-dependent manner (Supplementary Figure 4). These data demonstrate that a single copy of myr-*p110α* does not saturate the PI3K signaling pathway with regard to p-AKT levels and two copies of myr-*p110α* further activate PI3K signaling to a greater degree than that induced by a single copy of myr-*p110α* in mammary tumors, resulting in the increased rate of tumor initiation.

As the rate of tumor initiation was further influenced by the addition of *Kras*^*G12D*^ mutation into *p53*^*fl/fl*^;myr-*p110α*, which has been shown to signal primarily through the PI3K/AKT pathway,^30^ we assessed the levels of PI3K signaling activation in *p53*^*fl/fl*^;myr-*p110α* mammary tumors with or without *Kras*^*G12D*^ mutation. Mammary tumors from *p53*^*fl/fl*^;*Kras*^*G12D*^;myr-*p110α*^*wt/fl*^ and *p53*^*fl/fl*^;*Kras*^*G12D*^;myr-*p110α*^*fl/fl*^ had significantly increased levels of total p110α (1.3-fold) and p-AKT levels (1.3-fold), as compared with *p53*^*fl/fl*^;myr-*p110α*^*wt/fl*^ and *p53*^*fl/fl*^;myr-*p110α*^*fl/fl*^ mammary tumors as shown in Figures 3c and d. These findings show that PI3K signaling can be further activated by cooperation of myr-*p110α* with *Kras*^*G12D*^ mutation, and the rate of tumor initiation is strongly influenced by the level of PI3K activity.

Interestingly, the addition of two copies of myr-*p110α* also modestly but significantly accelerated tumor progression, as revealed by the survival curve (Figure 3e). Mammary tumors from *p53*^*fl/fl*^;*Kras*^*G12D*^;myr-*p110α*^*fl/fl*^ mice reached end point with a mean of 15.4±0.8 days, faster than *p53*^*fl/fl*^;*Kras*^*G12D*^;myr-*p110α*^*wt/fl*^ mice (18.4±0.5 days) and *p53*^*fl/fl*^;*Kras*^*G12D*^ mice (18.7±0.7 days).

### Addition of myr-*p110α* into *p53^fl/fl^*;*Kras^G12D^* double mutants modifies histologic phenotype

**Figure 4:**
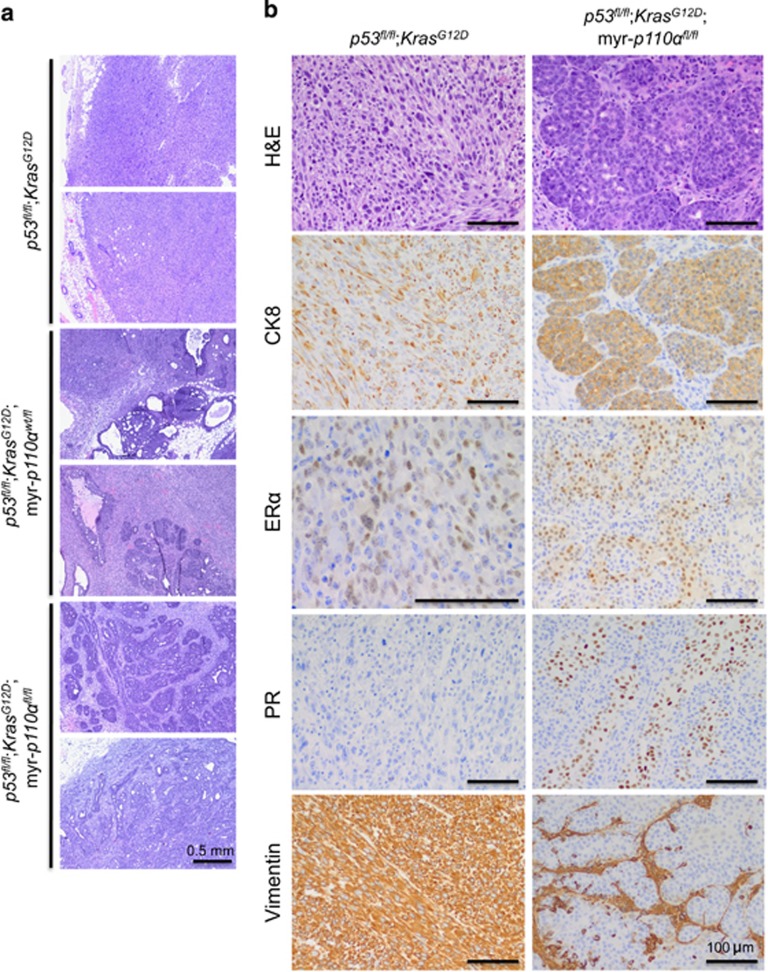
Addition of myr-*p110α* allele into *p53*^*fl/fl*^;*Kras*^*G12D*^ double mutants modifies tumor histology in a copy number-dependent manner. (**a**) Representative hematoxylin and eosin (H&E) sections of mammary tumors from *p53*^*fl/fl*^;*Kras*^*G12D*^, *p53*^*fl/fl*^;*Kras*^*G12D*^;myr-*p110α*^*wt/fl*^ and *p53*^*fl/fl*^;*Kras*^*G12D*^;myr-*p110α*^*fl/fl*^ mice showing increasing epithelial differentiation with addition of myr-*p110α* allele. Scale bar represents 0.5 mm and indicates scale for all images. (**b**) Comparison of histologic and immunohistochemical (CK8, ERα, PR and vimentin) phenotypes between *p53*^*fl/fl*^;*Kras*^*G12D*^ and *p53*^*fl/fl*^;*Kras*^*G12D*^;myr-*p110α*^*fl/fl*^ tumors. Representative H&E sections show solid sheets and fascicles of pleomorphic spindle cells in *p53*^*fl/fl*^;*Kras*^*G12D*^ tumor compared to the epithelial nests and glands present in *p53*^*fl/fl*^;*Kras*^*G12D*^;myr-*p110α*^*fl/fl*^ tumor. Both tumors are immunoreactive with CK8 and ERα the *p53*^*fl/fl*^;*Kras*^*G12D*^ tumor is PR negative and vimentin positive, whereas the *p53*^*fl/fl*^;*Kras*^*G12D*^;myr-*p110α*^*fl/fl*^ tumor is PR positive and vimentin negative. Scale bar represents 100 μm. Magnification is × 40 (**a**), × 400 (ERα staining of the *p53*^*fl/fl*^;*Kras*^*G12D*^ tumor in **b**) or × 200 (all remaining images in **b**).

Given the inherent heterogeneity of breast cancers, mammary tumors from the transgenic mice were characterized histologically (Figure 4). Mammary tumors from *p53*^*fl/fl*^;*Kras*^*G12D*^ mice were mostly poorly differentiated malignant neoplasms with sarcomatoid features. Mammary tumors from *p53*^*fl/fl*^;*Kras*^*G12D*^;myr-*p110α*^*wt/fl*^ also had a predominantly sarcomatoid histologic phenotype similar to *p53*^*fl/fl*^;*Kras*^*G12D*^ mammary tumors, but ~10–15% of tumor area was better differentiated with overt epithelial components including DCIS, papillary carcinoma and invasive ductal carcinoma.

Histological analysis of mammary tumors from *p53*^*fl/fl*^;*Kras*^*G12D*^;myr-*p110α*^*fl/fl*^ mice demonstrated an increase in tumors with a carcinoma phenotype including both DCIS and invasive ductal carcinoma (Figure 4a). It was also noted that there was an increase in benign epithelial proliferations including adenosis and papillomas. Some of the tumors had an admixed spindle cell component. As the copy number of myr-*p110α* doubled, the majority of *p53*^*fl/fl*^;myr-*p110α*^*fl/fl*^mammary tumors also developed a carcinoma phenotype, whereas *p53*^*fl/fl*^;myr-*p110α*^*wt/fl*^ mammary tumors developed dedifferentiated mixed tumors with sarcomatoid features and only focal areas of carcinoma (Supplementary Figure 5). These findings show that increased PI3K activity mediated by two copies of myr-*p110α,* when combined with either *p53*^*fl/fl*^ or *p53*^*fl/fl*^;*Kras*^*G12D*^, have the potential to skew mammary tumors from a sarcomatoid phenotype to a carcinoma phenotype more common in humans.

As part of the pathological characterization, tumors were stained for the following markers: CK5, CK14, CK8, ERα, PR, Her2/neu and vimentin. The majority of mammary tumors (invasive and *in situ* carcinoma, and benign proliferations) from *p53*^*fl/fl*^;*Kras*^*G12D*^;myr-*p110α*^*fl/fl*^ mice displayed strong CK8, ERα and PR immunostaining (Figure 4b). They were negative for vimentin (Figure 4b), whereas CK5 and CK14 immunostains confirmed the presence of myoepithlelial cells within DCIS and benign epithelial proliferations (data not shown). The sarcomatoid tumors from *p53*^*fl/fl*^;*Kras*^*G12D*^ mice were strongly positive for vimentin and showed variable expression of CK8, ERα and PR (Figure 4b). They were negative for CK5 and CK14. Tumors from *p53*^*fl/fl*^;*Kras*^*G12D*^;myr-*p110α*^*wt/fl*^ mice displayed greater immunostaining for CK8 compared to *p53*^*fl/fl*^;*Kras*^*G12D*^ mice (data not shown). None of the tumors from the three groups demonstrated positive (3+) Her2/neu immunostaining.

### Addition of myr-*p110α* into *p53^fl/fl^;Kras^G12D^* increases metastatic potential

**Figure 5:**
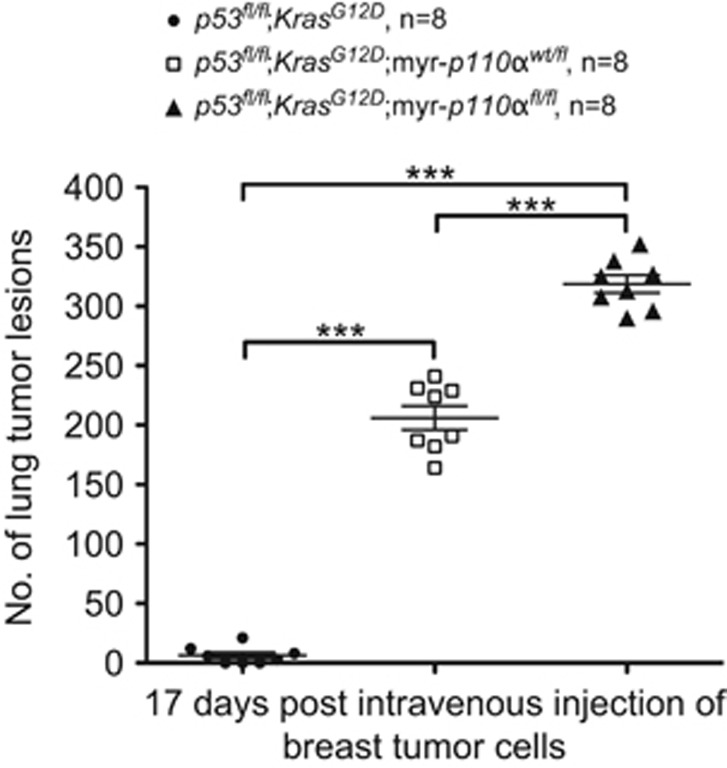
Combination of myr-*p110α* with *p53*^*fl/fl*^;*Kras*^*G12D*^ increases metastatic potential in a myr-*p110α* copy number-dependent manner. The graph showing the number of tumor lesions found in the lung surface 17 days after intravenous injection of breast tumor cells with different genotypes into the tail vein of syngeneic hosts. Error bars are the mean±s.e.m. Two-tailed unpaired Student's *t-*test was used for statistical analysis. ***P<0.001. In parallel, Mann–Whitney test (Wilcoxon rank-sum test) was used to reject the null hypothesis that there is no difference between groups (***Mann–Whitney *U-*value=0.0009). Data are representative of three independent experiments using *n*=8 per group with biological and technical replicates. Six- to eight-week-old syngeneic host mice were randomly assigned to generate similar numbers of subjects among groups.

To assay the ability of breast tumor cells to establish tumors outside the breast, we derived cells from *p53*^*fl/fl*^;*Kras*^*G12D*^, *p53*^*fl/fl*^;*Kras*^*G12D*^;myr-*p110α*^*wt/fl*^ and *p53*^*fl/fl*^;*Kras*^*G12D*^;myr-*p110α*^*fl/fl*^ mammary tumors, introduced them into the circulation by tail vein injection into syngeneic mice, and monitored for the formation of breast tumors in the lungs, as this method is considered to be a relevant model for breast cancer lung metastasis.^31^ 17 days after injection, mice injected with the *p53*^*fl/fl*^;*Kras*^*G12D*^;myr-*p110α*^*fl/fl*^ breast tumor cells produced significantly more identifiable tumors in the lungs (mean 318.6±7.4) compared with mice injected either with *p53*^*fl/fl*^;*Kras*^*G12D*^ breast tumor cells (mean 6.3±2.6) or *p53*^*fl/fl*^;*Kras*^*G12D*^;myr-*p110α*^*wt/fl*^ breast tumor cells (mean 206.1±10.0; Figure 5). This suggests that the myr-*p110α* promotes growth of metastatic tumors in a copy number-dependent manner.

### Addition of myr-*p110α* allele into *p53^fl/fl^;Kras^G12D^* double mutants accelerates tumor initiation in lung and decreases survival

One intentional aspect of the generation of this Cre-inducible mouse model and the utilization of Ad-cre virus is the ability to genetically modify different anatomic locations and potentially cause different tumor types. For the generation of lung cancer, we utilized injection of Ad-Cre virus into the trachea without surgery.

**Figure 6:**
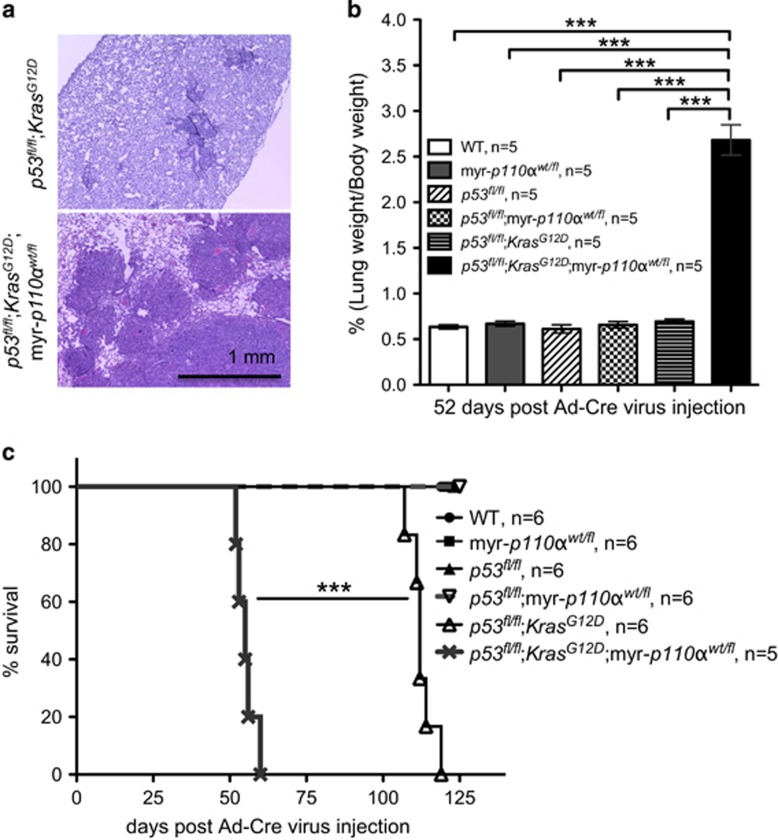
Addition of one copy of myr-*p110α* into *p53*^*fl/fl*^;*Kras*^*G12D*^ double mutants accelerates tumor development in lung. (**a**) Hematoxylin and eosin (H&E) staining of lungs dissected from *p53*^*fl/fl*^;*Kras*^*G12D*^ and *p53*^*fl/fl*^;*Kras*^*G12D*^;myr-*p110α*^*wt/fl*^ mice 52 days post Ad-Cre virus injection. Pictures of H&E staining were taken with × 40 magnification and are representative. Scale bar represents 1 mm and indicates scale for all images. (**b**) Bar graphs describing the weight of lung divided by body weight, 52 days after Ad-Cre virus injection. Error bars are the mean±s.e.m. Two-tailed unpaired Student's *t-*test was used for statistical analysis. ***P<0.001. (**c**) Kaplan–Meier survival curve scoring mice showing symptoms from lung tumor growth defined as any symptoms associated with illness. Log-rank (Mantel–Cox) test was used for statistical analysis. ***P<0.001. Data in **b** and **c** are representative of three independent experiments using *n*=5–6 per group.

Both *p53*^*fl/fl*^;*Kras*^*G12D*^ and *p53*^*fl/fl*^;*Kras*^*G12D*^;myr-*p110α*^*wt/fl*^ mice developed lung tumors with 100% penetrance. Histological analysis of lung tumors 52 days after intratracheal injection of Ad-Cre virus (when the first mice had symptoms of lung tumor growth) revealed high-grade adenocarcinomas that were largely disseminated throughout the lungs of *p53*^*fl/fl*^;*Kras*^*G12D*^;myr-*p110α*^*wt/fl*^ mice, whereas *p53*^*fl/fl*^;*Kras*^*G12D*^ mice developed only focal, small areas of adenocarcinoma (Figure 6a). Accordingly, lungs from *p53*^*fl/fl*^;*Kras*^*G12D*^;myr-*p110α*^*wt/fl*^ mice were enlarged and weighed two to three times more than lungs from either *p53*^*fl/fl*^;*Kras*^*G12D*^ mice or non-tumor-bearing mice, defining more tumor burdens (Figure 6b). These findings indicate that the addition of myr-*p110α* promotes lung tumor development. In contrast with breast cancer, the addition of one copy of myr-*p110α* into *p53*^*fl/fl*^;*Kras*^*G12D*^ did not change histology of the lung tumors. To further define the characteristics of the lung tumor development, mice were allowed to reach end point. The mean survival of *p53*^*fl/fl*^;*Kras*^*G12D*^;myr-*p110α*^*wt/fl*^ mice was 55±1.4 days, whereas the mean survival of *p53*^*fl/fl*^;*Kras*^*G12D*^ mice was 112±1.6 days, (Figure 6c). This finding was similar to the breast tumor experiments, in which survival time was cut in half by addition of one copy of myr-*p110α*. Overall, our observations demonstrate that the addition of constitutively active myr-*p110α* allele causes early tumor initiation in the lung, leading to early death related to lung tumors.

## Discussion

### Impact of constitutively active PI3K on tumor initiation and progression

It has recently been shown that cell signaling in many breast cancers is associated with an activated PI3K/AKT pathway.^32^ Notably, by evaluating differences in the p-AKT levels between mammary tumors harboring zero, one or two copies of myr-*p110α* allele combined with either *p53*^*fl/fl*^ or *p53*^*fl/fl*^;*Kras*^*G12D*^ mutants, our results proved that the levels of PI3K signaling activation are significantly increased and strongly correlated with the increased rate of tumor initiation in a myr-*p110α* copy number-dependent manner. These data demonstrated that higher levels of PI3K activity induced by myr-*p110α* addition have profound effects on tumor initiation. In addition, our results showed that the biological activity of PI3K signaling can be further activated by cooperation of myr-*p110α* with *Kras*^*G12D*^ mutation. Our data from the use of breast tumor cells also determined that the addition of myr-*p110α* allele into *p53*^*fl/fl*^;*Kras*^*G12D*^ mutants facilitates engraftment in the lungs, as an assay of metastatic potential, in a copy number-dependent manner. Overall, our findings provide experimental evidence that PI3K activating mutations associated with increased PI3K signaling activity accelerate the rate of tumor initiation and increase potential for metastasis.

Although *p53*^*fl/fl*^;*Kras*^*G12D*^;myr-*p110α*^*wt/fl*^ mice did not have increased growth rates of breast tumors as compared with *p53*^*fl/fl*^;*Kras*^*G12D*^ mice once tumors had developed, there was a modest but significant increase in the growth rate of tumors following initiation in *p53*^*fl/fl*^;*Kras*^*G12D*^ mice with two copies of myr-*p110α* as compared with *p53*^*fl/fl*^;*Kras*^*G12D*^ mice and *p53*^*fl/fl*^;*Kras*^*G12D*^mice with one copy of myr-*p110α*. One potential interpretation for these results is that sufficiently high levels of PI3K/AKT signaling activation also contribute to tumor growth rate.

### Impact of dosage of constitutively active PI3K on breast tumor phenotype

In our study, the addition of myr-*p110α* allele into *p53*^*fl/fl*^;*Kras*^*G12D*^ or *p53*^*fl/fl*^ that resulted in significantly increased PI3K/AKT signaling activation generated a carcinoma histologic phenotype in a myr-*p110α* copy number-dependent manner. The resulting tumors induced by the addition of myr-*p110α* allele resembled those that are commonly seen in the majority of human breast cancer. This was evident by the morphology of the invasive carcinoma, as well as by the increased presence of DCIS and benign epithelial proliferations. Thus, these findings suggest that levels of PI3K signaling might have a distinct impact on developing carcinoma phenotypes in humans. As the histological phenotype of tumors with two copies of myr-*p110α* was quite different than tumors with one copy, the increased growth rate induced by two copies could be attributed to the difference in tumor type generated by one versus two copies. One limitation of our study is that we did not define the relation between the spatial expression levels of myr-*p110α* or resulting p-AKT levels and histological features within the specific tumor areas. Additional studies are warranted to further evaluate this association between PI3K activity and tumor differentiation.

### Value of triple mutant transgenic mice for human cancer model

The value of genetically modified mouse models for cancer studies lies in the uniformity of the mouse background, the speed of cancer development enabling practical studies, and the similarity to human disease both genetically and histopathologically. The inherent heterogeneity of human breast cancer with variant histopathologic features even within a single tumor^33, 34^ creates an enormous challenge for understanding exact mechanisms of disease pathology. Our goal was to develop a breast cancer model that recapitulates the most common histopathological breast tumors, is temporally inducible and spatially controlled by the experimenter, and initiates and develops rapidly to facilitate practical experimentation.

Current mouse breast tumors driven by *PI3KCA* activating mutations mimic histopathologic phenotypes observed in human breast cancers;^17, 18, 19, 20, 35^ however, these single genetic mutation mouse models can take a year to develop breast tumors. Our mouse model that combines one or two copies of myr-*p110α* with *p53*^*fl/fl*^;*Kras*^*G12D*^ can resemble human breast cancer subtypes, as well as develop tumors early and grow quickly, leading to a shortened experimental timeline from tumor initiation to end stage.

Spontaneous additional mutations have been demonstrated to accumulate along with epigenetic events resulting in an increased level of genetic complexity in human cancer.^36^ The mouse model reported here is unique in that it harbors a *PI3KCA* (*p110α*) oncogene, which is frequently mutated in many human cancers,^3, 6^ along with the tumor suppressor gene *p53* deletion and a well-known oncogene, *Kras*^*G12D*^. These mutations are repeatedly altered in human cancers, so they represent relevant genetic alterations that can be used in developing models to closely mimic the pathological and biochemical features of cancers. In addition, this new mouse model is also able to rapidly generate cancers in other tissues by the application of Cre-recombinase with adenovirus as demonstrated by lung cancer generation, and it will be of scientific value to utilize this model for studying other tumor types. As the mutations require Cre-recombinase for manifestation, the triple mutation mouse lines can be maintained easily since breeding is not affected.

In summary, this novel triple mutation model system (1) is an autochthonous genetic model of human cancer, (2) provides controlled inducible expression of genetic changes spatially and temporally, (3) induces tumor subtypes matched histologically to human breast tumors, and (4) develops tumors very rapidly. The characteristics outlined should make this new genetically modified mouse model of significant value for understanding the molecular mechanisms underlying development of various tumors.

## Materials and methods

### Generation of experimental transgenic mice

The transgenic Cre-inducible myristoylated-*p110α* mice on C57BL/6 backgrounds were generated^22^ and intercrossed with Cre-inducible mice carrying either only *p53* deletion homozygote (*p53*^*fl/fl*^, which deletes exon 2 to exon 10 leading to null alleles of *p53* upon Cre-mediated excision)^37^ or *p53*^*fl/fl*^ and *Kras*^*G12D*^ mutations^29^ (*p53*^*fl/fl*^;*Kras*^*G12D*^, intercrossed) on C57BL/6 backgrounds. Conditional mutant mice with various genotypes were obtained and genotyped by PCR. The animal study protocol was reviewed and approved by the institutional animal care and use committee of Geisel School of Medicine at Dartmouth.

### *In vivo* intraductal delivery of Adeno-Cre virus into mammary duct

Six- to eight-week-old virgin females were anesthetized by isofluorane/oxygen inhalation. The calcium phosphate precipitate containing 2.5 × 10^7^ plaque-forming unit of Ad-Cre virus was injected into the teat canal of fourth inguinal mammary duct using a Hamilton syringe with 33-G needle.^23^ Mice were monitored daily for a palpable/visible tumor formation. Tumor surface area was calculated by the formula: tumor size (mm^2^)=length (mm) × width (mm), after caliper measurements.

### Hematoxylin and eosin stain

Paraffin-embedded tissues after fixation with 10% buffered formalin (15740-01, Electron Microscopy Sciences, Hatfield, PA, USA) were sectioned at 6 μm. Deparaffinized sections were stained with hematoxylin and eosin following the manufacturer's protocol and mounted with Permount (SP15-100, Fisher Scientific, Grand Island, NY, USA). Images were obtained on an Olympus BX5.1 or BX4 microscope (Olympus, Waltham, MA, USA), and captured using Image Pro software (Media Cybernetics, Rockville, MD, USA).

### Immunohistochemistry

Tumors were paraffin-embedded after fixation in 10% buffered formalin and sectioned at 6 μm. Sections were immunostained using standard manufacturer's protocols at the Dartmouth-Hitchcock Medical Center Laboratory for Clinical Genomics and Advanced Technology with the following antibodies: anti-CK5 (ab52635, Abcam, Cambridge, MA, USA), anti-CK14 (RB-9020, Thermo Scientific, Fremont, CA, USA), anti-CK8 (ab53280, Abcam), anti-estrogen receptor alpha (SC-542, Santa Cruz Biotechnology, Santa Cruz, CA, USA), anti-PR (RM-9102, Thermo Scientific), anti-HER2/neu (MA5-15050, Thermo Scientific) and anti-vimentin (ab92547, Abcam). Images were obtained using an Olympus BX5.1 or BX41 microscope and Image Pro software. Hematoxylin and eosin-stained and immunostained slides were reviewed by a breast pathologist (JDM).

### Protein preparation and western blot

Mammary tumors were lysed in ice-cold lysis buffer containing 50 mM Tris–HCl (pH 7.4), 150 mM NaCl, 1% Triton X-100, 0.1% SDS, 1 mM NaVO_4_, protease inhibitor (11836170001, Roche, Indianapolis, IN, USA) and phosphatase inhibitor cocktail (P5726, Sigma-Aldrich, St Louis, MO, USA). An amount of 20 μg proteins were separated on 9% SDS-polyacrylamide gel electrophoresis gels and transferred to Immobilon-P membranes (IPVH00010, Millipore, Darmstadt, Germany). According to the standard protocols, the membranes were immunoblotted with following primary antibodies: anti-p110α (4249, Cell Signaling Technology, Beverley, CA, USA), anti-AKT1/2/3 (4691, Cell Signaling Technology), anti-p-AKT1/2/3 Ser473 (4060, Cell Signaling Technology), anti-p85α (sc-1637, Santa Cruz Biotechnology), anti-PTEN (9559, Cell Signaling Technology), anti-p-PTEN Ser380/Thr382/Thr383 (sc-101789, Santa Cruz Biotechnology), anti-GFP (sc-9996, Santa Cruz Biotechnology) and horseradish peroxidase-conjugated anti-β-Actin (ab49900, Abcam). This was followed by incubation with horseradish peroxidase-conjugated anti-rabbit (sc-2313, Santa Cruz Biotechnology) or anti-mouse (sc-2060, Santa Cruz Biotechnology) secondary antibody. Immunoreactive proteins were detected by chemiluminescence ECL (sc-2048, Santa Cruz Biotechnology), quantified using ImageLab software (Bio-Rad, Hercules, CA, USA) and normalized with respect to β-actin expression.

### Preparation of breast tumor cells and assay of metastatic potential

The cells were derived from culturing large numbers of dissociated breast tumor cells in complete DMEM over several passages (less than five). The metastatic potential of breast tumor cells was tested by intravenous injection of 0.5 × 10^6^ cells into the tail vein of syngeneic mice. The number of tumor lesions over 1.5 mm in diameter on the lungs was counted macroscopically after stained with 0.05% 1,9-dimethyl-methylene blue.

### *in vivo* intratracheal delivery of Adeno-Cre virus into the lung

Six- to eight-week-old mice were anesthetized by intraperitoneal injection of avertin (0.3 mg/g body weight). The Exel Safelet IV catheter was inserted into the mouth and slid into the trachea, and the calcium phosphate precipitate containing 2.5 × 10^7^ plaque-forming unit of Ad-Cre virus was injected through the opening of the catheter using a 0.5-ml syringe. Mice were monitored for clinical symptoms of poor health, such as lethargy, panting/respiratory distress and/or decreased mobility.

### Mouse embryonic fibroblasts (MEFs) preparation

MEFs were derived and cultured as previously described.^22^

### Ad-Cre virus infection of MEFs

60–70% confluent MEFs at passage number 2–3 were infected at multiplicity of infection 100:1 (virus:cell) with adenovirus-expressing Cre-recombinase (Ad5CMVCre, University of Iowa, Gene Transfer Vector Core) in DMEM supplemented with 2% fetal bovine serum for 6 h. The infection efficiency was confirmed by flow cytometry of eGFP-positive cells to ensure that >85% cells expressed the myr-*p110α*.

### Anchorage-independent cell growth assay *in vitro*

A total of 2000 pooled transfected MEFs were resuspended in a top layer of 0.35% agar-containing DMEM and plated onto a bottom layer of 0.5% agar-containing DMEM in 12-well plates in triplicate. After 30 days, the colonies were stained with 0.005% crystal violet and counted macroscopically. Minimum diameter of the colonies was 50 μm.

### *In vivo* tumor formation assay

Total 1 × 10^6^ pooled transfected MEFs were injected intradermally into six- to eight-week-old immunocompromised NOD/scid IL2 gamma chain KO (NSG, NOD.Cg-*Prkdc*^*scid*^ *Il2rg*^*tm1Wjl*^/SzJ) mice. Mice were monitored daily to detect a palpable/visible tumor.

### Statistical analysis

Two-tailed unpaired Student's *t-*test and the Mann–Whitney test were used for comparison between experimental groups analyzed for anchorage-independent cell growth assay, metastatic potential assay and western blots. For experiments involving a comparison between three or more distinct groups, one-way analysis of variance test was used. Statistics were calculated as non-parametric Log-rank (Mantel–Cox) analysis of Kaplan–Meier for censored survival and tumor-free data. Data analysis was performed using Prism 5.0 software (GraphPad software, La Jolla, CA, USA). Graphs in figures denoted statistical significance of *P<0.05, **P<0.01, and ***P<0.001. *P*>0.05 was considered non-significant (ns).
