# Supplementary material for: Constitutively activated PI3K accelerates tumor initiation and modifies histopathology of breast cancer
Source: Oncogenesis. 2016 Oct 31;5(10):e267–. doi: 10.1038/oncsis.2016.65 (PMC5141269; doi:10.1038/oncsis.2016.65)
Supplement: Supplementary Data [file oncsis201665x1.pdf]

## Supplementary Data

**Supplementary Figure 1.** Cre-inducible *Rosa26*-myr-*p110α* knock-in allele with or without Cre-recombinase.

**Supplementary Figure 2.** Expression of myr-*p110α* with deletion of *p53* in mouse embryonic fibroblasts induces transformation *in vitro* and *in vivo.*

**Supplementary Figure 3.** Frequency of mutations in three genes, *PIK3CA, p53,* and *Kras*, in human breast cancers.

**Supplementary Figure 4.** Addition of myr-*p110α* elevates the levels of PI3K signaling activation in a copy number-dependent manner.

**Supplementary Figure 5.** Increased gene dosage of myr-*p110α*, when combined with *p53* deletion, modifies tumor subtype to carcinoma.
