# Supplementary material for: Constitutively activated PI3K accelerates tumor initiation and modifies histopathology of breast cancer
Source: Oncogenesis. 2016 Oct 31;5(10):e267–. doi: 10.1038/oncsis.2016.65 (PMC5141269; doi:10.1038/oncsis.2016.65)
Supplement: Supplementary Figure 1 [file oncsis201665x2.pdf]

Supplementary Figure 1

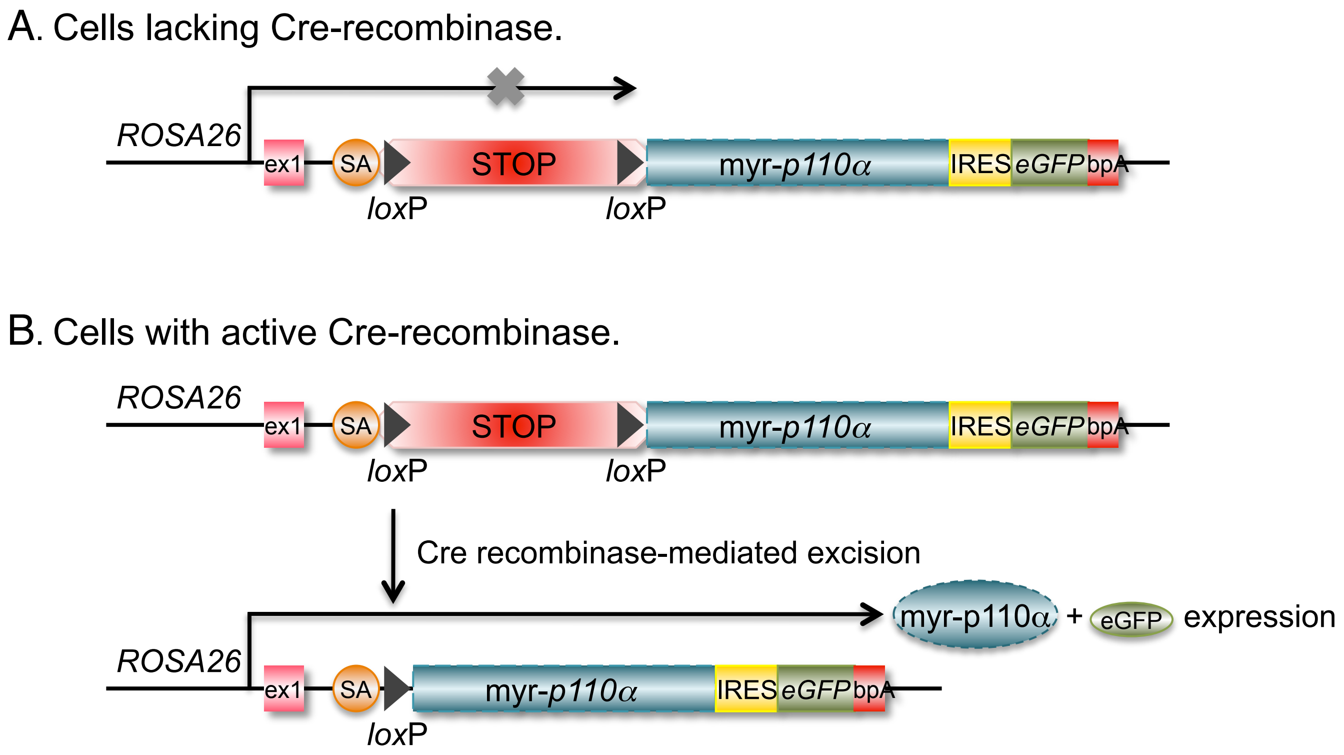

Supplementary Figure 1. Cre-inducible *Rosa26*-myr-*p110α* knock-in allele with or without Cre-recombinase. Schematic diagrams of the expression construct inserted into *Rosa26* locus. (A) In cells lacking Cre-recombinase, the STOP cassette blocks expression of myr-*p110α* from the endogenous *Rosa26* promoter. (B) In cells with active Cre-recombinase, Cre-mediated removal of the STOP cassette links *Rosa26* exon 1 to myr-*p110α*. As the endogenous *Rosa26* transcripts are not translated into protein, the first start codon in the myr-*p110α* is used for initiation of translation, and thus myr-*p110α* is expressed. Abbreviations: ex1, exon 1; SA, splice acceptor; Myr-*p110α*, myristoylated-*p110α*; IRES, internal ribosome entry site; eGFP, enhanced green fluorescent protein; bpA, BGH poly A terminator.
