# Supplementary material for: Constitutively activated PI3K accelerates tumor initiation and modifies histopathology of breast cancer
Source: Oncogenesis. 2016 Oct 31;5(10):e267–. doi: 10.1038/oncsis.2016.65 (PMC5141269; doi:10.1038/oncsis.2016.65)
Supplement: Supplementary Figure 2 [file oncsis201665x3.pdf]

Supplementary Figure 2

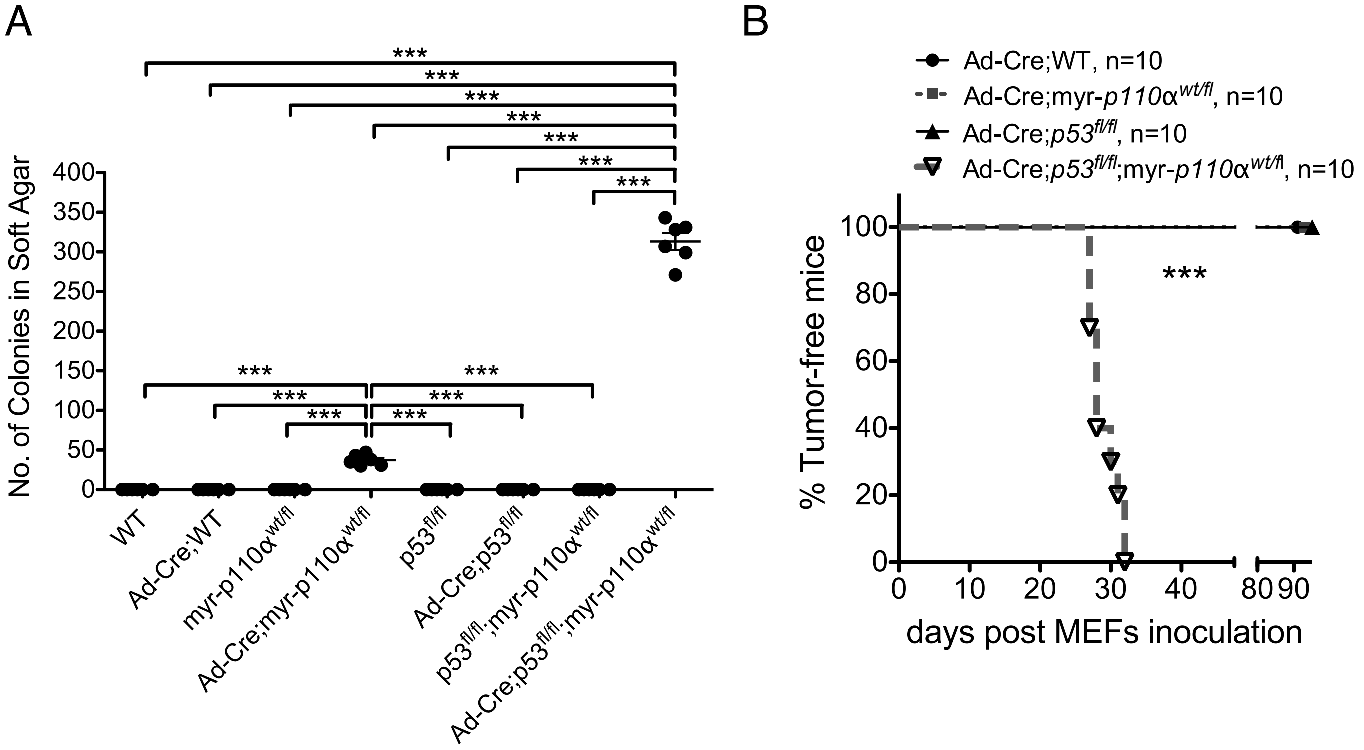

Supplementary Figure 2. Expression of myr-*p110α* with deletion of *p53* in mouse embryonic fibroblasts induces transformation *in vitro* and *in vivo*. (A) Graph describing the number of colonies in soft agar after 30 days of culture with each pool of stably transfected MEFs at passage number 4-5. Error bars are the mean ± SEM. Two-tailed unpaired Student's *t*-test was used for statistical analysis. ***$P < 0.001$. Data are representative of 3 independent experiments using n=6 per group. (B) Kaplan-Meier survival curve demonstrating dermal tumor onset in NSG hosts post intradermal injection of each pool of stably transfected MEFs at passage number 4-5. The date of tumor onset was defined as the first recognition of palpable/visible tumor ranges 10-12 mm^2 size. Log-rank (Mantel-Cox) test was used for statistical analysis. ***$P < 0.001$. Data are representative of 3 independent experiments using n=10 per group.
