# Supplementary material for: Constitutively activated PI3K accelerates tumor initiation and modifies histopathology of breast cancer
Source: Oncogenesis. 2016 Oct 31;5(10):e267–. doi: 10.1038/oncsis.2016.65 (PMC5141269; doi:10.1038/oncsis.2016.65)
Supplement: Supplementary Figure 3 [file oncsis201665x4.pdf]

Supplementary Figure 3

A

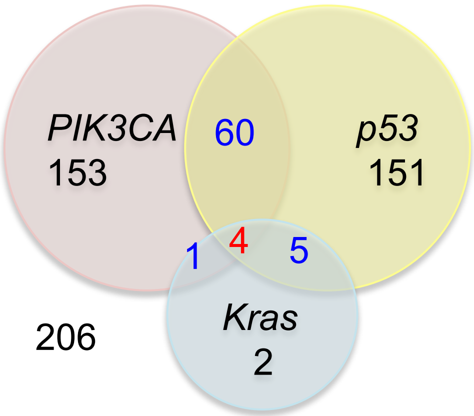

B

Table. The mutual exclusivity between *PIK3CA* (*p110α*), *p53,* and *Kras* in human breast cancers defined by the odds ratio.

GENE	*PIK3CA*	*p53*	*Kras*
PIK3CA	—	0.5541126 (****p*=0.0007)	1.197183 (*p*=0.7698)
p53		—	3.463022 (**p*=0.0461)
Kras			—

No association (0.5 < Odds Ratio < 2)
Tendency toward co-occurrence (2 < Odds Ratio < 10)

Supplementary Figure 3. Frequency of mutations in three genes, *PIK3CA, p53,* and *Kras*, in human breast cancers. (A) Diagram showing number of cases with mutations of three genes, *PIK3CA, p53,* and *Kras* in human breast cancers. (B) Table demonstrating mutual exclusivity between *PIK3CA, p53,* and *Kras* in human breast cancers. The odds ratio is indicated with *p* value that was derived via Fisher's Exact test. **P* < 0.05, ***P* < 0.01, and ****P* < 0.001. Data are based on published cancer genomics data collected from 582 breast cancer patients (COSMIC, http://cancer.sanger.ac.uk/cancergenome/projects/cosmic/).
