# Supplementary material for: Constitutively activated PI3K accelerates tumor initiation and modifies histopathology of breast cancer
Source: Oncogenesis. 2016 Oct 31;5(10):e267–. doi: 10.1038/oncsis.2016.65 (PMC5141269; doi:10.1038/oncsis.2016.65)
Supplement: Supplementary Figure 4 [file oncsis201665x5.pdf]

Supplementary Figure 4

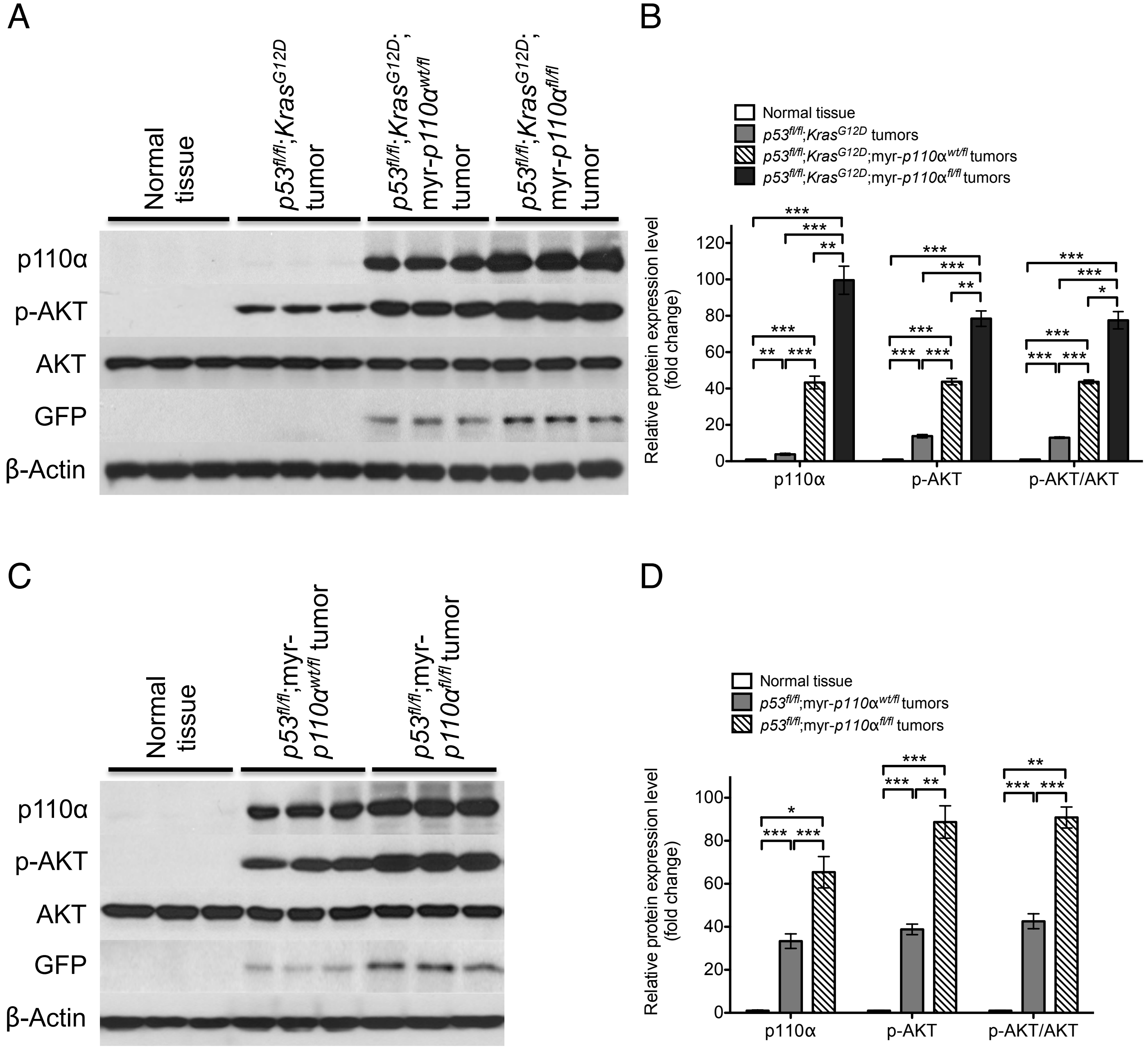

Supplementary Figure 4. Addition of myr-*p110α* elevates the levels of PI3K signaling activation in a copy number-dependent manner. (A) Western blot analysis of p110α, p-AKT, AKT, and GFP with normal mammary tissue and mammary tumors from $p53^{fl/fl}$;$Kras^{G12D}$, $p53^{fl/fl}$;$Kras^{G12D}$;myr-$p110\alpha^{wt/fl}$, and $p53^{fl/fl}$;$Kras^{G12D}$;myr-$p110\alpha^{fl/fl}$ mice. (B) The graphs of Western blot signals of p110α, p-AKT, and p-AKT/AKT ratio shown in (A). (C) Western blot

analysis of p110α, p-AKT, AKT, and GFP with normal mammary tissue and mammary tumors from *p53*$^{fl/fl}$;myr-*p110α*$^{wt/fl}$ and *p53*$^{fl/fl}$;myr-*p110α*$^{fl/fl}$ mice. (D) The graphs of Western blot signals of p110α, p-AKT, and p-AKT/AKT ratio shown in (C). The Western blot signals quantified and normalized with respect to β-Actin protein expression. Mean fold increase compared with normal mammary tissue lysates from a non-Cre harboring age-matched female, was calculated after normalization. Error bars are presented as the mean ± SEM. Two-tailed unpaired Student's *t*-test was used for statistical analysis. *$P < 0.05$, **$P < 0.01$, and ***$P < 0.001$. Expression of myr-*p110α* was confirmed by the detection of GFP in mammary tumors. Data are representative of 3 independent experiments with biological and technical replicates.
