# Supplementary material for: Constitutively activated PI3K accelerates tumor initiation and modifies histopathology of breast cancer
Source: Oncogenesis. 2016 Oct 31;5(10):e267–. doi: 10.1038/oncsis.2016.65 (PMC5141269; doi:10.1038/oncsis.2016.65)
Supplement: Supplementary Figure 5 [file oncsis201665x6.pdf]

Supplementary Figure 5

A

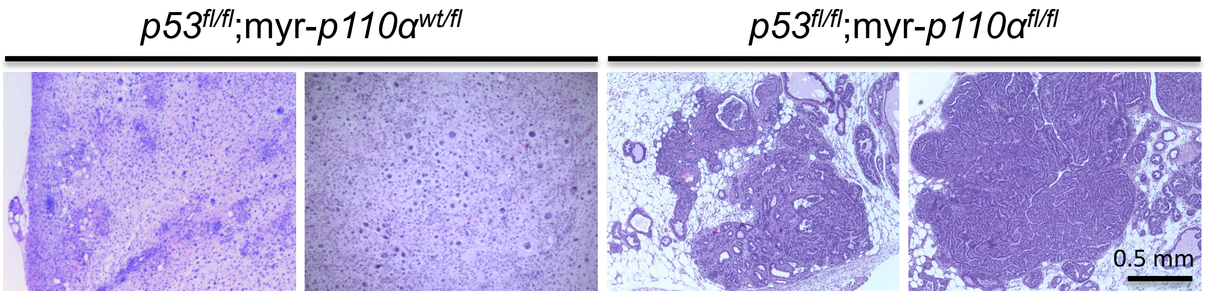

Supplementary Figure 5. Increased gene dosage of myr-*p110α*, when combined with *p53* deletion, modifies tumor subtype to carcinoma. Hematoxylin and eosin (H&E) staining of mammary tumors from $p53^{fl/fl}$;myr-$p110\alpha^{wt/fl}$ and $p53^{fl/fl}$;myr-$p110\alpha^{fl/fl}$ females. Pictures of H&E staining were taken with X40 magnification. Scale bar represents 0.5 mm and indicates scale for all images. Pictures are representative.
